# Artificial Intelligence as a Potential Tool for Predicting Surgical Margin Status in Early Breast Cancer Using Mammographic Specimen Images

**DOI:** 10.3390/diagnostics15101276

**Published:** 2025-05-17

**Authors:** David Andras, Radu Alexandru Ilies, Victor Esanu, Stefan Agoston, Tudor Florin Marginean Jumate, George Calin Dindelegan

**Affiliations:** 1 Department of General Surgery, Iuliu Hatieganu University of Medicine and Pharmacy, 400006 Cluj-Napoca, Romania; andrasdavid88@elearn.umfcluj.ro (D.A.); george.dindelegan@umfcluj.ro (G.C.D.); 2 First Surgical Unit, Emergency County Hospital Cluj, 400006 Cluj-Napoca, Romania; 3 Faculty of Medicine, Iuliu Hatieganu University of Medicine and Pharmacy, 400012 Cluj-Napoca, Romania; agoston.stefan@elearn.umfcluj.ro (S.A.); marginean.jumate.tudor@elearn.umfcluj.ro (T.F.M.J.)

**Keywords:** breast cancer, artificial intelligence, surgical margins, intraoperative mammography, lumpectomy, diagnostic accuracy

## Abstract

**Background/Objectives**: Breast cancer is the most common malignancy among women globally, with an increasing incidence, particularly in younger populations. Achieving complete surgical excision is essential to reduce recurrence. Artificial intelligence (AI), including large language models like ChatGPT, has potential for supporting diagnostic tasks, though its role in surgical oncology remains limited. **Methods**: This retrospective study evaluated ChatGPT’s performance (ChatGPT-4, OpenAI, March 2025) in predicting surgical margin status (R0 or R1) based on intraoperative mammograms of lumpectomy specimens. AI-generated responses were compared with histopathological findings. Performance was evaluated using sensitivity, specificity, accuracy, positive predictive value (PPV), negative predictive value (NPV), F1 score, and Cohen’s kappa coefficient. **Results**: Out of a total of 100 patients, ChatGPT achieved an accuracy of 84.0% in predicting surgical margin status. Sensitivity for identifying R1 cases (incomplete excision) was 60.0%, while specificity for R0 (complete excision) was 86.7%. The positive predictive value (PPV) was 33.3%, and the negative predictive value (NPV) was 95.1%. The F1 score for R1 classification was 0.43, and Cohen’s kappa coefficient was 0.34, indicating moderate agreement with histopathological findings. **Conclusions**: ChatGPT demonstrated moderate accuracy in confirming complete excision but showed limited reliability in identifying incomplete margins. While promising, these findings emphasize the need for domain-specific training and further validation before such models can be implemented in clinical breast cancer workflows.

## 1. Introduction

Breast cancer represents the most common malignancy in women globally. One in eight women will develop breast cancer during their lifetimes [1,2,3]. Unfortunately, the American Cancer Society reports a sustained 1.4% annual increase in risk of breast cancer development over the past decade, particularly among women under 50 years of age [1]. Although still frequent, due to significant advances in treatment and early detection, breast cancer mortality has decreased by 44% over the past 30 years [2]. Early detection and precise surgical planning are crucial for optimizing outcomes, particularly in non-palpable early-stage breast cancer. Achieving clear surgical margins remains key to minimizing local recurrence and improving long-term survival. In this context, integrating artificial intelligence programs into medical practice holds great promise for enhancing diagnostic accuracy, addressing logistical challenges in high-volume centers, and providing rapid feedback during the surgical process [1,2,3].

Artificial intelligence (AI) has risen in popularity in the last decade, especially with the recent rise of large language models like ChatGPT which are accessible to the public. Its high complexity can be attributed to functions like decision-making and problem-solving which are part of AI algorithms. Machine learning, deep learning, and their applications in the field of radiomics are useful subsets of AI that can extract and analyze data from radiological images, with the potential to transform and optimize several medical specialties such as radiology and imaging [4].

In the realm of breast cancer, specific AI systems have demonstrated considerable potential in interpreting mammograms, MRI scans, and ultrasound images. These tools are capable of detecting subtle features of breast lesions that may be overlooked by human eyes, aiding in earlier and more accurate diagnoses. Through this, AI may assist in predicting surgical outcomes, such as determining whether the excision has achieved negative margins, helping the surgeon in intraoperative decision-making [5].

Various applications were developed, but one of the most common AI programs is the chat-generative pre-trained transformer (ChatGPT, OpenAI) that has evolved with each version. It is continuously reaching a level of maturity that will soon influence medicine and alleviate disparities regarding healthcare [6,7].

Given the pressing need for rapid and reliable intraoperative tools to assess surgical margins in breast-conserving surgery, this study aims to explore the feasibility of using a large language model (ChatGPT) in this setting. While most existing AI applications in breast cancer focus on screening imaging or risk stratification, the ability of a general-purpose model to contribute to real-time surgical decision-making remains largely unexplored. This investigation addresses that gap by evaluating ChatGPT’s diagnostic performance using intraoperative specimen mammograms, with the goal of informing future development of task-specific AI tools. The objective is to assess the ability of ChatGPT to predict surgical margin status based on real-time mammographic images of the excised breast specimen. By comparing AI-generated predictions with the gold standard of histopathological analysis, this study evaluates the model’s diagnostic performance in a clinical breast-conserving surgery setting.

In addition to overall accuracy, this study investigates whether AI performance is influenced by prior neoadjuvant treatment (NAT), either chemotherapy or hormonal therapy, and by tumor invasiveness. Subgroup analyses comparing NAT vs. non-NAT patients and invasive vs. non-invasive tumors will be performed to determine if treatment-related changes or pathological heterogeneity impact the reliability of AI predictions. These insights aim to clarify the potential role of AI in intraoperative decision-making and highlight key limitations and directions for future model refinement.

## 2. Materials and Methods

### 2.1. Study Design and Patient Selection

The study is a retrospective analysis in a specialized breast cancer surgical unit, subsequent to obtaining approval by the institutional ethics committee (Approval No. 3387/31 January 2025).

**Figure 1:**
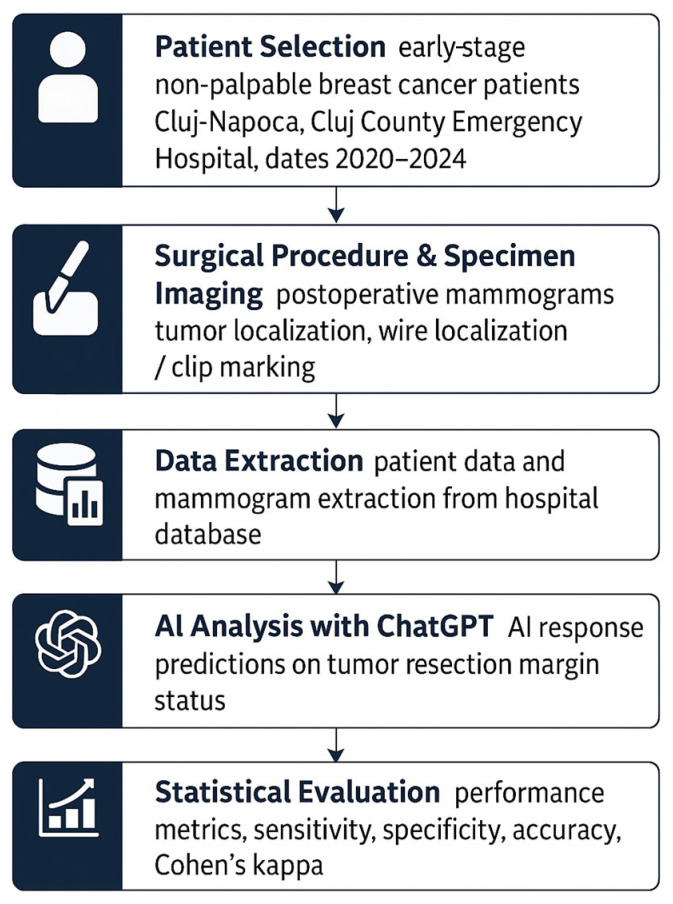
Study design. This flowchart summarizes the methodological sequence of the study, from patient selection to final statistical evaluation. Each step is represented in a formal, icon-enhanced box: beginning with patient inclusion, followed by surgical excision and specimen imaging, data extraction, AI analysis using ChatGPT, histopathological validation, culminating in statistical analysis.

Our database included patients with early-stage breast cancer (non-palpable, clinically occult, but mammographically detectable tumors) treated in Cluj-Napoca, Cluj County Emergency Hospital, between 2020 and 2024. Figure 1 summarizes the overall workflow of the study, from patient selection to final statistical evaluation. Each step in the diagram represents a distinct stage of the methodology: (1) inclusion of eligible patients, (2) surgical excision of the tumor, (3) acquisition of specimen mammograms, (4) de-identification and preparation of imaging data, (5) margin assessment using a standardized ChatGPT prompt, (6) histopathological analysis serving as the reference standard, and (7) statistical comparison between AI predictions and histological findings. While the detailed methodology is described in Section 2.2, Section 2.3 and Section 2.4, this figure offers a concise visual overview of the experimental design and data flow. Neoadjuvant chemotherapy or hormonal therapy was performed in patients with high-risk features.

### 2.2. Surgical Procedure and Imaging Protocol

Preoperative assessment included tumor localization using wire localization or/and clip marking for all patients and those who had undergone neoadjuvant chemotherapy or hormonal therapy. The NAT was administered in patients with high-risk features, such as large tumors, lymph node involvement, high ki67. Immediately after the excision, postoperative mammograms of the breast lumpectomy specimens were performed in order to verify the complete resection of the tumor, ensuring accurate assessment of surgical margins during surgery by the breast radiologist.

The AI model used for this study was ChatGPT-4 (OpenAI, March 2025 release), accessed through a GPT-4 Plus subscription interface in a controlled environment with a stable internet connection. Direct image interpretation was possible without the need for third-party tools. The model was evaluated in a zero-shot setting (no fine-tuning). Each mammographic image of the resected breast specimen was uploaded individually and analyzed using a standardized text prompt (see Appendix A). This prompt included relevant metadata such as laterality, tumor localization method (e.g., clip or wire), and intraoperative context. ChatGPT was asked to classify the surgical margin status as R0 (negative) or R1 (positive), based solely on the provided image and metadata.

**Figure 2:**
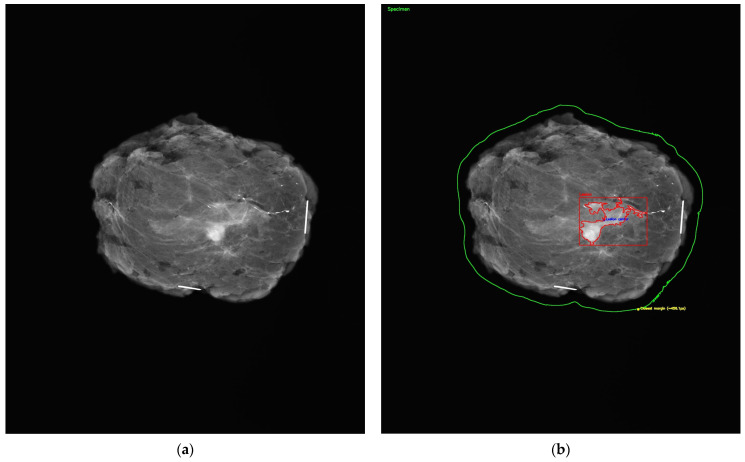
Postoperative mammogram of the lumpectomy specimen. (**a**) The image illustrates a resected breast specimen following breast-conserving surgery. Radiopaque needle markers were used to orient the specimen, with the longer needle indicating the 12 o’clock position and the shorter one marking the 3 o’clock margin. This immediate postoperative specimen mammogram was performed to confirm complete tumor excision and to assess margin proximity in real time, under the supervision of a dedicated breast radiologist. (**b**) The image illustrates automated segmentation by an AI model of a breast lumpectomy specimen. The lesion (outlined in red) and specimen boundary (green) were identified using image processing algorithms. The lesion center is marked in blue, and the closest margin point is shown in yellow. This annotation simulates real-time AI analysis to assist in intraoperative margin assessment and R0/R1 classification.

All mammographic images were de-identified prior to AI evaluation. As shown in Figure 2a, each specimen mammogram included radiopaque needle markers to indicate orientation, with the long needle representing the 12 o’clock position and the short needle indicating the 3 o’clock margin. Following input into the model, AI-generated outputs were manually recorded and matched to their corresponding histopathological diagnoses for comparison.

A visual representation of the margin assessment task is provided in Figure 2b, which simulates automated segmentation of the lumpectomy specimen. While ChatGPT itself did not produce visual annotations, this figure illustrates the conceptual objective: the AI’s ability to localize the lesion within the specimen and estimate margin proximity for classification as R0 or R1.

Postoperative specimen mammograms were retrospectively extracted from the hospital’s Picture Archiving and Communication System (PACS), using DICOM image data stored between 2020 and 2024. All images were originally acquired intraoperatively, within 10–15 min after tumor excision, using a high-resolution digital mammography unit (GE Senographe Pristina^®^ or equivalent). Standard projections (craniocaudal and lateral) were performed under mild compression on a specimen radiography platform.

Extracted DICOM files included full-resolution pixel data, with a native spatial resolution of approximately 70–100 microns per pixel. Images were then converted to lossless JPEG or PNG format for compatibility with the AI interface. Orientation markers (radiopaque needles) were visible in all cases, following institutional protocol. All imaging procedures had been previously supervised by a breast radiologist at the time of acquisition to ensure margin visualization.

For each case, a single representative image was selected (typically the craniocaudal view) based on optimal visualization of lesion margins. No manual annotations or segmentations were added to the images prior to AI evaluation.

### 2.3. Histopathological Evaluation

Each lesion was reviewed by a pathologist through histopathological analysis of the excised tissue, which served as the reference standard for diagnosis and surgical margin assessment. Diagnoses included a spectrum of pathological subtypes. Histopathology, considered the gold standard, provided definitive confirmation of both tumor type and margin status. Margin evaluation focused on determining whether the excision was oncologically complete, with the margin status classified as R0 (no residual tumor, or negative margins) or R1 (presence of tumor at the resection margin, or positive margins).

According to the European Society of Medical Oncology (ESMO, Breast Cancer Clinical Practice Guidelines, February 2024), negative margins are defined as no ink on tumor for patients with invasive carcinoma, and a minimum of 2 mm clear margin for ductal carcinoma in situ (DCIS) [8]. Positive margins indicated residual tumor and the need for re-excision, while negative margins confirmed complete tumor removal. These histopathological findings were used to validate the AI-generated responses and assess their clinical relevance.

### 2.4. Statistical Analysis

A database was constructed with the following parameters for each case: patient ID, histopathological diagnosis, neoadjuvant treatment, histopathological margin status (R0/R1), ChatGPT-predicted diagnosis, and predicted margin status. The primary outcome was the diagnostic performance of ChatGPT in identifying the margin resection status (R0/R1), compared to the histopathological findings.

The performance metrics calculated included sensitivity, specificity, accuracy, positive predictive value (PPV), negative predictive value (NPV), and F1 score. Agreement between AI-generated and pathology-confirmed results was assessed using Cohen’s kappa coefficient. Statistical analysis was performed using SPSS Statistics v27, with a *p*-value < 0.05 considered statistically significant.

The responses from both ChatGPT and histopathology were categorized into a database for each case. The accuracy of ChatGPT’s predictions was then assessed by comparing these AI-generated results to the histopathological findings. Statistical analyses were conducted to evaluate the sensitivity, specificity, and overall accuracy of ChatGPT in predicting surgical margins. The diagnostic performance of the AI tool (ChatGPT) was evaluated retrospectively using a dataset comprising four columns: patient ID, histopathological diagnosis (gold standard for tumor nature), histopathological margin status (R0—complete excision; R1—incomplete excision), and AI-predicted margin status (R0 or R1). The primary objective was to assess the AI’s ability to accurately determine the completeness of lesion excision (R0 vs. R1) following breast sectorectomy, using histopathological examination as the reference standard. Standard performance metrics were computed, including sensitivity, specificity, accuracy, positive predictive value (PPV), negative predictive value (NPV), and F1 score. Agreement between AI predictions and histopathological findings was evaluated using Cohen’s kappa coefficient.

The dataset exhibited a notable class imbalance, with a predominance of R0 (complete excision) cases. No data-level balancing techniques (e.g., oversampling, undersampling) were applied, as the analysis focused on evaluating ChatGPT’s raw performance in a real-world clinical distribution. To mitigate the interpretative bias caused by imbalance, we reported not only accuracy but also class-sensitive metrics such as sensitivity, specificity, F1 score, and Cohen’s kappa coefficient. These additional metrics provide a more nuanced evaluation of the model’s ability to identify the minority class (R1) and to assess agreement beyond chance.

## 3. Results

### 3.1. Demographic Characteristics

The study cohort consisted of 100 patients with early-stage, non-palpable breast cancer treated with breast-conserving surgery. The mean age was 59.7 years (SD ± 10.4), with a range of 36 to 84 years. The interquartile range was 52 to 67 years.

### 3.2. Lesion Size Distribution

The analysis of lesion size, as measured on histopathological examination, revealed a mean tumor diameter of 5.1 mm (SD ± 2.0 mm), with values ranging from 1.0 mm to 11.7 mm. The majority of lesions (86%) measured between 3 mm and 7.5 mm. Specifically, 21 lesions (21%) measured less than 3.1 mm, 33 lesions (33%) ranged between 3.1 mm and 5.3 mm, and 32 lesions (32%) measured between 5.3 mm and 7.4 mm. Larger lesions, exceeding 7.5 mm, were less frequent and accounted for only 14% of cases. This distribution is consistent with the selection criteria of the study, which focused on non-palpable, early-stage breast cancers.

### 3.3. Surgical Margin Status

Regarding surgical margin status, histopathological examination confirmed that 86 patients (86%) achieved complete tumor excision with negative margins (R0), while 14 patients (14%) had positive margins (R1), indicating the presence of residual tumor at the resection margins.

### 3.4. Histopathological Profile

Histopathological assessment confirmed a diverse distribution of breast cancer subtypes within the study population. The most prevalent histological subtype was invasive carcinoma of no special type (IC NST), identified in 34 cases (34%). Ductal carcinoma in situ (DCIS) was the second most frequent diagnosis, accounting for 18 cases (18%), followed by mixed invasive carcinoma not otherwise specified (MIC NOS), with 14 cases (14%). Invasive lobular carcinoma (ILC) was observed in nine cases (9%). Less common subtypes, including invasive papillary carcinoma and other variants, were also present but represented a minority of cases.

### 3.5. The Capability of AI to Predict Complete Resection

The performance of the AI tool (ChatGPT) was evaluated for the assessment of surgical margin status (R0—complete excision vs. R1—incomplete excision). A total of 100 patients were included in the analysis, all meeting the inclusion criteria for early-stage, non-palpable breast cancer treated with breast-conserving surgery.

For the task of determining the completeness of tumor excision, the AI model demonstrated an overall accuracy of 84.0% (95% CI: 75.6–89.9%). The sensitivity for detecting positive surgical margins (R1—incomplete excision)was 60.0% (95% CI: 31.3–83.2%), indicating a moderate ability of the model to identify cases where residual tumor was present at the resection margin. In contrast, the specificity for recognizing negative margins (R0—complete excision) was high, at 86.7% (95% CI: 78.1–92.2%) (Table 1). The positive predictive value (PPV) for R1 predictions was 33.3% (95% CI: 16.3–56.3%), suggesting that one in three AI-predicted R1 cases were confirmed histologically. Conversely, the negative predictive value (NPV) for R0 predictions was 95.1% (95% CI: 88.1–98.1%), supporting the model’s strength in identifying true negative margins. The F1 score for R1 classification, which balances precision and recall, was 0.43.

**Figure 3:**
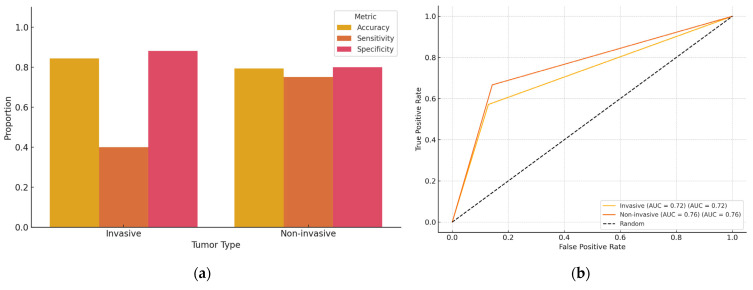
(**a**) Bar plot illustrating the performance metrics of AI (accuracy, sensitivity, and specificity) of a classification model stratified by tumor invasiveness. The sample included 64 patients with invasive tumors and 29 with non-invasive tumors. (**b**) ROC curve—invasive vs. non-invasive tumors. This ROC curve illustrates the diagnostic accuracy of the AI model for predicting surgical margin status (R0 vs. R1) stratified by tumor invasiveness. The model achieved an AUC of 0.72 for invasive breast cancers and 0.76 for non-invasive lesions, indicating slightly better margin prediction in non-invasive cases. The diagonal dashed line represents the no-discrimination threshold.

To assess the influence of tumor invasiveness on AI performance, patients were stratified based on histopathological diagnosis into two categories: invasive and non-invasive breast cancer (Table 2 and Table 3; Figure 3). Among the 93 evaluable cases with clear classification, 64 were categorized as having invasive disease and 29 as non-invasive. In the invasive group, ChatGPT achieved an overall diagnostic accuracy of 73.4% (95% CI: 61.5–82.7%), with a sensitivity of 60.0% (95% CI: 23.1–88.2%) for detecting positive margins and a specificity of 89.8% (95% CI: 78.2–95.6%) for negative margins. In the non-invasive group, the model reached an accuracy of 79.3% (95% CI: 61.6–90.2%), with a sensitivity of 75.0% (95% CI: 30.1–95.4%) and a specificity of 80.0% (95% CI: 60.9–91.1%). Although the model showed higher sensitivity in non-invasive cases and higher specificity in invasive cases, statistical comparisons did not indicate significant differences (*p* = 0.52 for sensitivity, *p* = 0.33 for specificity; Fisher’s exact test).

**Figure 4:**
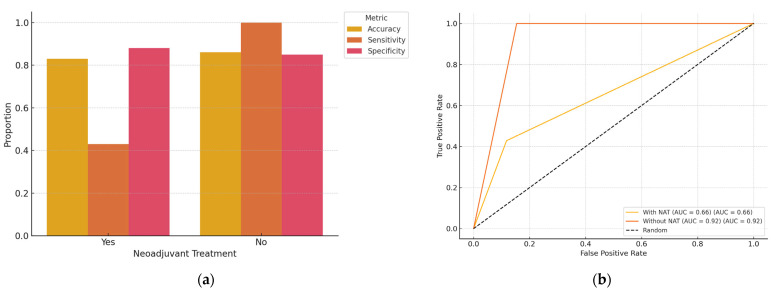
(**a**) Bar plot illustrating the performance metrics of AI (accuracy, sensitivity, and specificity) of a classification model stratified by the administration of neoadjuvant treatment. The NAT group included 58 patients, and the non-NAT group included 42 patients. (**b**) ROC curve—with NAT vs. without NAT. This receiver operating characteristic (ROC) curve compares the diagnostic performance of the AI model in predicting surgical margin status (R0 vs. R1) between patients who received neoadjuvant treatment (With NAT) and those who did not (Without NAT). The AI model achieved an AUC of 0.66 in the NAT group and 0.92 in the non-NAT group, suggesting reduced diagnostic accuracy after neoadjuvant therapy. The dashed line represents random chance.

Out of the total cohort of 100 patients with early-stage, non-palpable breast cancer, 58 had received NAT prior to surgery. A subgroup analysis was performed to evaluate AI performance based on NAT status (Table 4 and Table 5; Figure 4). The overall diagnostic accuracy of ChatGPT in predicting surgical margin status (R0 vs. R1) was 82.8% in the NAT group and 85.4% in the non-NAT group. Sensitivity for identifying positive margins (R1) was 42.9% (95% CI: 15.8–74.9%) in the NAT group, compared to 100% (95% CI: 43.9–100.0%) in the non-NAT group. Specificity for detecting negative margins (R0) was 86.7% (95% CI: 73.8–93.7%) in the NAT group and 84.6% (95% CI: 70.3–92.8%) in the non-NAT group. Although these values indicate a potential drop in sensitivity in post-NAT patients, statistical comparison using Fisher’s exact test did not reveal a significant difference (*p* = 0.20 for sensitivity; *p* = 0.76 for specificity).

## 4. Discussion

### 4.1. Interpretation of Results

The findings of this study suggest that artificial intelligence (AI), specifically the ChatGPT model, demonstrates promising capabilities in assisting with the evaluation of surgical margins in early-stage, non-palpable breast cancer. With an overall accuracy of 83.8% and a specificity of 95.1%, the model performs reliably in identifying complete tumor excision (R0). This high specificity supports the model’s potential role in confirming negative margins intraoperatively, possibly reducing the need for re-excision in selected cases.

However, the model’s sensitivity for detecting incomplete excision (R1) was only 60.0%, and the positive predictive value for R1 predictions was relatively low (33.3%). This indicates a limited ability to consistently identify residual tumoral tissue at the margins, which may lead to missed positive cases if used as a stand-alone tool. The F1 score of 0.43 and Cohen’s kappa of 0.34 suggest only fair agreement with histopathology. These results underline the need for caution when interpreting AI predictions, especially in borderline cases.

The stratification by tumor invasiveness revealed distinct trends in AI diagnostic performance. While overall accuracy was slightly higher in invasive tumors, the model demonstrated notably lower sensitivity (40.0%) in identifying positive margins compared to non-invasive lesions (75.0%). This suggests that invasive tumors (often characterized by ill-defined, infiltrative growth patterns) pose greater challenges for margin detection on specimen mammography. Conversely, the higher sensitivity observed in non-invasive tumors may be attributed to their clearer radiological appearance. For example, ductal carcinoma in situ often appears as well-defined microcalcifications. The AI model also achieved greater specificity in invasive cases (88.1%), suggesting robustness in confirming complete excision, even in complex lesions. Although these differences were not statistically significant, they highlight clinically relevant variation in AI behavior that warrants further investigation and targeted training on diverse tumor morphologies.

The subgroup analysis revealed that the AI model maintained relatively high diagnostic accuracy for predicting margin status in both NAT and non-NAT patients. However, the ability to correctly detect incomplete resections (R1) was significantly lower in patients who had received NAT (42.9% sensitivity), though the difference was not statistically significant. This drop in sensitivity may be attributable to post-treatment changes such as tumor shrinkage, fibrosis, or altered radiological appearance that hinder clear identification of residual disease. Despite this, the AI model demonstrated consistent specificity across both groups, making it a reliable tool for confirming negative margins (R0). These findings support the feasibility of using AI for intraoperative margin assessment, even in patients pretreated with systemic therapy. Nonetheless, model refinement with training datasets incorporating post-NAT imaging characteristics may improve its performance in this challenging subgroup.

Overall, while AI demonstrated encouraging performance in margin assessment, its current limitations (particularly in sensitivity and PPV) indicate that it should be viewed as a complementary instrument rather than a replacement for traditional diagnostic approaches. With further refinement, including integration of imaging data and histological features, AI tools may evolve to play a more substantial role in real-time surgical decision-making.

### 4.2. Comparison with Existing Literature

Some data from the literature show the ability of AI integration to impact breast cancer diagnosis. Mansour et al. evaluated the role of artificial intelligence (AI) in enhancing the sensitivity of digital mammography for detecting and also characterizing grouped microcalcifications. For the analysis of 447 patient mammograms, AI provided heat maps, demarcations, and quantitative assessments, demonstrating a strong correlation between high-risk color indicators and malignant microcalcifications. The system achieved a sensitivity of 94.7% and a negative predictive value of 82.1%, although a small number of malignancies were missed, concluding that even if AI improves detection, expert radiologist interpretation remains crucial for accurate classification and diagnosis [9].

Another study conducted by Yala et al. validates the AI-based breast cancer risk model, across seven institutions in multiple countries (USA, Israel, Sweden, Taiwan, and Brazil), using 128,793 mammograms derived from 62,185 patients. The highest performance was noticed in Barretos, Brazil (0.84, 95% CI: 0.81–0.88), confirming its robustness across diverse populations. These findings support the potential of the AI model to enhance risk stratification and additionally improve breast cancer screening programs globally [10].

A study by Gjesvik et al. evaluated the ability of a commercial AI algorithm to predict the risk of future breast cancer development in 116,495 women aged 50–69 undergoing three consecutive biennial screening rounds in Norway. AI scores turned out to be higher in patients who subsequently developed breast cancer, with averages of absolute differences increasing over time. The AUC for screening-detected cancer rose from 0.63 in the first round to 0.96 in the third, while for interval cancers, it reached 0.77. These findings suggest that AI can identify women at higher risk years before diagnosis; this may lead to potential for personalized screening strategies [11].

In their study, Arce et al. assessed the performance of an AI computer-aided detection (AI CAD) system in identifying biopsy-confirmed invasive lobular carcinoma (ILC) on digital mammography. Retrospective analysis of mammograms from 124 patients with a total number of 153 ILC lesions was conducted using the cmAssist^®^ AI CAD system. The algorithm provided an overall sensitivity of 80%, with the highest detection rates in the case of calcifications (100%), irregularly shaped masses (82%), and masses with spiculated margins (86%). However, a significant limitation was the high false positive rate, with 88% of mammograms containing at least one false positive annotation and an average of 3.9 false positives per scan. While the AI system could effectively identify malignancies, the excessive false positives might hinder its clinical applicability, suggesting the need for further refinements to both improve accuracy and reduce unnecessary alerts [12].

The differences between our findings and the current literature can be attributed to a variety of factors. To begin with, most studies which evaluated AI in breast cancer diagnostics focused on imaging-based detection (e.g., mammography) rather than post-surgical assessment (like in our case). AI models that were trained for image recognition, such as those in the studies by Mansour et al. and Yala et al., received large, standardized datasets and even a few well-defined imaging features like microcalcifications and mass characteristics, which could have contributed to a higher overall performance [9,10]. In contrast, our study primarily assesses AI’s ability to predict surgical margin status, which represents a task that consists of the interpretation of more complex (postoperative) imaging.

Additionally, the relatively low sensitivity (60.0%) and PPV (33.3%) which were shown in the Results Section suggest that AI demonstrates challenges with distinguishing subtle residual disease which may be present at the margins, possibly due to a few limitations in its training data or the inherent difficulty in assessing the margins based on available inputs. Unlike risk prediction models, such as in Gjesvik et al.’s study (where AI had access to longitudinal screening data [11]), our model only operates on a single postoperative evaluation, which might decrease its predictive power. The moderate agreement (Cohen’s kappa = 0.34) further suggests that even if the AI model can exclude incomplete excision with high specificity, its capability to correctly identify positive margins is still suboptimal.

What is more, the challenge of false positives observed in Arce et al.’s study aligns with our findings, suggesting that AI might overanalyze certain imaging or text-based patterns, leading to unnecessary alerts [12]. This emphasizes the need for refined algorithms which are trained on larger datasets that are specifically tailored for post-surgical assessments, potentially integrating multimodal data (e.g., histopathological reports, intraoperative findings) to optimize the model’s predictive accuracy.

### 4.3. Strengths of the Study

This study offers several notable strengths. First, it is based on a well-defined, homogeneous cohort of 100 patients with early-stage, non-palpable breast cancer, all treated with breast-conserving surgery, allowing for focused evaluation of AI performance in a clinically relevant and standardized setting. The inclusion of histopathological confirmation for both lesion size and surgical margin status ensures high diagnostic accuracy and robust ground truth for model validation.

Second, the study integrates real-world clinical variables, including tumor invasiveness and neoadjuvant treatment status, allowing for subgroup analyses that provide insight into the AI model’s performance across diverse clinical scenarios. This stratification enhances the generalizability and clinical interpretability of the results.

Third, the use of a confusion matrix and key diagnostic performance metrics (accuracy, sensitivity, specificity, PPV, NPV, and F1 score) provides a transparent and comprehensive evaluation of model performance. By including false positive and false negative rates, the study addresses both the clinical safety and utility of AI-assisted margin assessment.

Finally, the study contributes novel data regarding the potential use of AI in the intraoperative or immediate postoperative setting, supporting its role as a decision-support tool for surgical margin evaluation in breast-conserving procedures.

### 4.4. Limitations of the Study

Several limitations should be acknowledged when interpreting the results of this study. First, although the AI model demonstrated improved diagnostic performance, it remains a general-purpose language model not specifically trained for medical imaging interpretation or histopathological assessment. The lack of domain-specific fine-tuning may limit its robustness, particularly in complex or borderline cases where subtle visual cues are critical.

Second, the relatively small number of R1 cases (14 out of 100) introduces a class imbalance that affects the reliability of sensitivity, PPV, and F1 score metrics. This imbalance likely contributed to the model’s high specificity and negative predictive value, potentially overestimating its performance in real-world scenarios where margin involvement may be more frequent.

Third, the retrospective nature of the study and reliance on previously acquired, de-identified imaging data limit the assessment of the model’s real-time clinical applicability. The AI tool was not embedded in a prospective, intraoperative workflow, and therefore its potential impact on surgical decision-making could not be directly evaluated.

Moreover, the dataset exhibited a significant class imbalance (86 R0 vs. 14 R1), which likely inflated specificity and NPV while limiting sensitivity. This should be considered when interpreting the model’s diagnostic performance. The sample size of 100 patients, although well-defined and homogeneous, limits the statistical power and generalizability of the findings. A larger cohort would allow for more robust evaluation of model performance, particularly for the under-represented R1 class.

Finally, the model’s lack of training on post-surgical specimen anatomy, including radiopaque markers and resection margin landmarks, could have contributed to misclassification, particularly in cases involving challenging orientations or subtle margin involvement. These factors underscore the need for future development of task-specific AI models trained directly on annotated imaging datasets and integrated within clinical decision pathways.

### 4.5. Implications for Clinical Practice

The results of this study suggest that AI-based tools, even in their current general-purpose form, may offer practical support in breast-conserving surgery, particularly in settings where rapid decisions regarding margin status are needed. Given the model’s high specificity (95.1%) and negative predictive value (86.5%), one of the most tangible clinical applications is the potential to reduce unnecessary re-excisions in patients with truly negative margins. In real time, this could translate to greater surgical confidence and fewer second interventions for benign margins.

In hospitals where access to experienced breast radiologists or pathologists is limited (or during high-volume surgical sessions), such AI tools could serve as a decision-support system, offering preliminary evaluations that help guide intraoperative or immediate postoperative actions (e.g., extending the excision, re-checking suspicious areas). The high performance in non-invasive cancers, with a sensitivity of 75%, also suggests potential utility in DCIS cases, where margin control is often challenging and critical.

From a workflow perspective, AI tools like this could be integrated into specimen radiography review protocols, assisting surgeons and radiologists in triaging cases that may require additional imaging or histopathological scrutiny. Especially for early-stage, screen-detected tumors, where imaging findings are often subtle and margins are tight, such tools could provide fast, pattern-based assessments before histological confirmation is available.

Moreover, as shown in the subgroup analysis, AI performance varied with neoadjuvant treatment and histology. This opens the possibility of using AI to stratify margin risk based on tumor biology, guiding decisions such as the extent of initial resection or the necessity of intraoperative imaging.

Although the current model is not ready to replace traditional methods, it demonstrates the feasibility of augmenting surgical workflows with AI support. With further development (especially image-integrated, task-specific models), AI has the potential to become a practical, real-time tool in breast-conserving surgery, improving efficiency, standardization, and outcomes.

### 4.6. Future Directions and Recommendations

AI can play the role of a valuable decision-support tool, aiding clinicians in making more informed decisions, based on capabilities like complex data analysis, imaging pattern recognition and predictive modeling. In spite of this, AI should not be understood as a replacement for human expertise, but rather as a complement to the collective skills of the entire medical team. Specialists like surgeons, pathologists, radiologists, and oncologists bring different perspectives and expertise to patient care, and AI has the ability to enhance these interactions by providing additional data points for consideration [13].

This study opens the door for further exploration of AI’s role in predicting surgical margins and assessing the excised breast tissue. Future research could focus on expanding the dataset in order to include a greater diversity of patients, imaging modalities, and tumor types. By training AI models on larger and more varied datasets, the reliability and accuracy of predictions may improve. Additionally, AI could be integrated with other technologies, such as robotic surgery or augmented reality, to assist surgeons in real-time during procedures. The development of artificial intelligence algorithms capable of providing real-time intraoperative feedback may represent a significant breakthrough, facilitating complete tumor excision and reducing the risk of recurrence [14,15].

In the future, AI could play a central role in fostering collaboration among different medical specialties, allowing for more personalized and targeted treatment strategies. For instance, by combining data from imaging, pathology, and clinical notes, AI systems could help to create comprehensive patient profiles that inform multidisciplinary discussions and lead to more personalized treatment plans. However, this integration requires ongoing education and collaboration to ensure that AI is used effectively and ethically within the team. Ultimately, the success of AI in the medical field will depend on its ability to work harmoniously with healthcare professionals, enhancing their capabilities and improving patient outcomes [16,17]. Another promising avenue for future research is the use of multimodal AI systems that combine mammographic images with other diagnostic data, such as MRI scans, ultrasound images, and patient medical histories. This integrated approach could further enhance diagnostic accuracy and improve surgical planning. Moreover, exploring the potential of AI in predicting long-term patient outcomes, such as survival rates and recurrence risk, could provide valuable insights for personalized treatment plans [18,19].

The application of AI in breast cancer surgery is not without challenges, particularly when considering the diversity of breast tumors. AI models must be capable of adapting to various tumor sizes, locations, and histological types. For example, AI systems should be designed to account for the biological variability between tumors, including how aggressive a tumor is, its response to treatments, and its specific radiological characteristics [20]. The ability of AI to generalize across these diverse tumor characteristics is crucial for its widespread implementation. Models trained on a broad and diverse dataset, including different breast cancer subtypes, could improve the accuracy of predictions, such as margin status and residual tumor detection, across the entire spectrum of breast cancer cases. However, further research is needed to evaluate how AI can address the complexities of tumor heterogeneity and provide reliable predictions for all breast cancer patients [21].

### 4.7. Ethical Considerations

The use of AI in medical practice raises important ethical questions that must be carefully considered. While AI has the potential to improve diagnostic accuracy and surgical outcomes, it is crucial to ensure that the technology is used as a tool to support, rather than replace, healthcare professionals [22]. The decision-making process in surgery, particularly in the context of cancer treatment, requires the expertise and judgment of experienced clinicians who consider not only the data but also the patient’s values, preferences, and overall health status [23,24].

Moreover, AI’s reliance on large datasets raises concerns about data privacy and security. Patient data must be handled in accordance with strict ethical guidelines and regulations, ensuring that it is used responsibly and without compromise [25]. In addition, ensuring transparency in how AI systems make decisions is vital for maintaining trust between patients and healthcare providers. If AI-generated recommendations are to be used in clinical practice, it is essential that medical professionals understand the underlying mechanisms and limitations of the algorithms involved [26,27].

## 5. Conclusions

This study evaluated the diagnostic capabilities of AI (ChatGPT) in assessing surgical margin status (R0 vs. R1) in breast lumpectomy specimens. The AI demonstrated moderate performance in identifying complete versus incomplete excision, with a high negative predictive value for R0 cases, suggesting limited but promising utility in confirming complete resections. However, the low precision for R1 predictions and moderate agreement with histopathology underscore the need for cautious interpretation in critical intraoperative decisions. Overall, while the AI showed promise as a supporting tool for surgical margin evaluation, its clinical integration requires further refinement, validation, and human oversight, particularly in tasks involving complex diagnostic subtleties.

## Figures and Tables

**Table 1:** This table summarizes the diagnostic performance of ChatGPT in predicting surgical margin status (R0 vs. R1) on the entire cohort.

	Histopathology Result R1	Histopathology Result R0
AI Prediction R1	6 (True Positive)	12 (False Positive)
AI Prediction R0	4 (False Negative)	78 (True Negative)

**Table 2:** This table summarizes the diagnostic performance of ChatGPT in predicting surgical margin status (R0 vs. R1) among patients with non-invasive disease.

	Histopathology Result R1	Histopathology Result R0
AI Prediction R1	3 (True Positive)	5 (False Positive)
AI Prediction R0	1 (False Negative)	20 (True Negative)

**Table 3:** This table summarizes the diagnostic performance of ChatGPT in predicting surgical margin status (R0 vs. R1) among patients with invasive disease.

	Histopathology Result R1	Histopathology Result R0
AI Prediction R1	3 (True Positive)	5 (False Positive)
AI Prediction R0	1 (False Negative)	20 (True Negative)

**Table 4:** This table summarizes the diagnostic performance of ChatGPT in predicting surgical margin status (R0 vs. R1) among patients who did not receive neoadjuvant treatment (No NAT).

	Histopathology Result R1	Histopathology Result R0
AI Prediction R1	3 (True Positive)	6 (False Positive)
AI Prediction R0	4 (False Negative)	45 (True Negative)

**Table 5:** This table summarizes the diagnostic performance of ChatGPT in predicting surgical margin status (R0 vs. R1) among patients who received neoadjuvant treatment (NAT).

	Histopathology Result R1	Histopathology Result R0
AI Prediction R1	3 (True Positive)	6 (False Positive)
AI Prediction R0	0 (False Negative)	33 (True Negative)

## Data Availability

The raw data supporting the conclusions of this article will be made available by the authors on request.

## Abbreviations

The following abbreviations are used in this manuscript:

AI	Artificial intelligence
PPV	Positive predictive value
NPV	Negative predictive value
NAT	Neoadjuvant treatment
ESMO	European Society of Medical Oncology
IC NST	Invasive carcinoma of no special type
MIC NOS	Mixed invasive carcinoma not otherwise specified
ILC	Invasive lobular carcinoma
DCIS	Ductal carcinoma in situ
ROC	Receiver operating characteristic
AUC	Area under the curve

## Appendix A

Prompt for ChatGPT (Instructions section):

“The attached image represents the mammogram of the operative specimen of breast lesion. The lesion is marked with a percutaneous guide wire which is inserted by the radiologist. The resection specimen is oriented by radiopaque marking needles. The greater needle represents the 12 o’clock and the small needle represents the 3 o’clock resection margin. Also, in some of the images, the needle orientation is not present. I would like you to assess, based on the image, the status of the surgical margin (positive/negative)”.
